# Genome of *Rhizobium leucaenae* strains CFN 299^T^ and CPAO 29.8: searching for genes related to a successful symbiotic performance under stressful conditions

**DOI:** 10.1186/s12864-016-2859-z

**Published:** 2016-08-02

**Authors:** Ernesto Ormeño-Orrillo, Douglas Fabiano Gomes, Pablo del Cerro, Ana Tereza Ribeiro Vasconcelos, Carlos Canchaya, Luiz Gonzaga Paula Almeida, Fabio Martins Mercante, Francisco Javier Ollero, Manuel Megías, Mariangela Hungria

**Affiliations:** 1 Universidad Nacional Agraria La Molina, Av. La Molina s/n La Molina, Lima, Peru; 2 Embrapa Soja, C.P. 231, 86001-970 Londrina, Paraná Brazil; 3 CAPES, SBN, Quadra 2, Bloco L, Lote 06, Edifício Capes, 70.040-020 Brasília, Federal District Brazil; 4 Departamento de Microbiología, Facultad de Biología, Universidad de Sevilla, Avda. Reina Mercedes, 6 Apdo Postal, 41012 Sevilla, Spain; 5 Laboratório Nacional de Computação Científica (LNCC), Labinfo, Rua Getúlio Vargas 333, 25651-071 Petrópolis, Rio de Janeiro Brazil; 6 Department Biochemistry, Genetics and Immunology, Faculty of Biology, University of Vigo, 36310 Vigo, Spain; 7 Embrapa Agropecuária Oeste, C.P. 449, 79804-970 Dourados, Mato Grosso do Sul Brazil

**Keywords:** Stress tolerance, Biological nitrogen fixation, Nodulation, Nod factors, Symbioses, Secretion systems

## Abstract

**Background:**

Common bean (*Phaseolus vulgaris* L.) is the most important legume cropped worldwide for food production and its agronomic performance can be greatly improved if the benefits from symbiotic nitrogen fixation are maximized. The legume is known for its high promiscuity in nodulating with several *Rhizobium* species, but those belonging to the *Rhizobium tropici* “group” are the most successful and efficient in fixing nitrogen in tropical acid soils. *Rhizobium leucaenae* belongs to this group, which is abundant in the Brazilian “Cerrados” soils and frequently submitted to several environmental stresses. Here we present the first high-quality genome drafts of *R. leucaenae*, including the type strain CFN 299^T^ and the very efficient strain CPAO 29.8. Our main objective was to identify features that explain the successful capacity of *R. leucaenae* in nodulating common bean under stressful environmental conditions.

**Results:**

The genomes of *R. leucaenae* strains CFN 299^T^ and CPAO 29.8 were estimated at 6.7–6.8 Mbp; 7015 and 6899 coding sequences (CDS) were predicted, respectively, 6264 of which are common to both strains. The genomes of both strains present a large number of CDS that may confer tolerance of high temperatures, acid soils, salinity and water deficiency. Types I, II, IV-pili, IV and V secretion systems were present in both strains and might help soil and host colonization as well as the symbiotic performance under stressful conditions. The symbiotic plasmid of CPAO 29.8 is highly similar to already described tropici pSyms, including five copies of *nodD* and three of *nodA* genes. *R. leucaenae* CFN 299^T^ is capable of synthesizing Nod factors in the absence of flavonoids when submitted to osmotic stress, indicating that under abiotic stress the regulation of *nod* genes might be different.

**Conclusion:**

A detailed study of the genes putatively related to stress tolerance in *R. leucaenae* highlighted an intricate pattern comprising a variety of mechanisms that are probably orchestrated to tolerate the stressful conditions to which the strains are submitted on a daily basis. The capacity to synthesize Nod factors under abiotic stress might follow the same regulatory pathways as in CIAT 899^T^ and may help both to improve bacterial survival and to expand host range to guarantee the perpetuation of the symbiosis.

**Electronic supplementary material:**

The online version of this article (doi:10.1186/s12864-016-2859-z) contains supplementary material, which is available to authorized users.

## Background

Biological nitrogen fixation (BNF) is a key process for global N inputs, and greatly contributes to avoidance of soil-nutrient impoverishment, to recovery of fertility of degraded areas, and also to reduce the emission of greenhouse gases related to application of chemical fertilizers. The greatest contribution of BNF occurs by means of symbiotic diazotrophic bacteria—collectively known as rhizobia—associated with several legumes, partially or fully supplying plant’s N needs [1, 2].

Considering the nutritional needs of growing populations, particularly in developing countries, undoubtedly common bean (*Phaseolus vulgaris* L.) represents the most important legume cropped for food purposes. A strategic goal of research with the legume is to maximize BNF through the selection and/or breeding of both plant and rhizobial genotypes [2–4]. Common bean is well known for its high promiscuity in associating with a variety of rhizobial species [5, 6], and in acid tropical soils of South America strains related to the *Rhizobium tropici* “group” represent the most efficient microsymbionts [6–9].

*R. tropici* was first described in 1991 [5], and its main features include its ability to nodulate both common bean and leucaena (*Leucaena* spp.), its high tolerance of stressful environmental tropical conditions, and higher genetic stability of the symbiotic plasmid in comparison to other common bean rhizobia (e.g. [4, 5, 9–11]). Although *R. tropici* is thought to have originated in South America [5], it is also found in Europe, Australia, Africa, and North and Central America [9]. The species has also been isolated from other host legumes, including *Gliricidia* spp., *Acaciella angustissima*, *Mimosa caesalpiniifolia*, *Bolusanthus* spp., *Aspartium* spp., and *Lotus tenuis* [9]. In addition to this promiscuity, also intriguing is the capacity of *R. tropici* to synthesize a variety of nodulation (Nod) factors [12, 13], even in the absence of plant molecular signals [12–15].

Despite long-standing reports of high phenetic and genetic diversity of strains within the *R. tropici* species [5], it was only two decades later that a group of strains named as *R. tropici* type A was split from the type-B group and reclassified as *Rhizobium leucaenae* [6]. The new species shares typical properties with *R. tropici*, such as high genetic stability of the symbiotic plasmid and tolerance of stressful environmental conditions [6].

*R. tropici*, *R. leucaenae* and two other new species previously classified as *R. tropici*—*Rhizobium freirei* [7] and *Rhizobium paranaense* [8]—encompass strains very effective in fixing nitrogen with common bean, representing an important source of inoculants for sustainable agriculture in the tropics (e.g. [4, 9]). However, the only available genomes of the group are of *R. tropici* CIAT 899^T^ and *R. freirei* PRF 81^T^ [11]. Here we present two genomes of *R. leucaenae*, including the type strain CFN 299^T^ and strain CPAO 29.8, very effective in fixing nitrogen with common bean. Our main objective was to search for genes that could help to explain the successful symbiotic performance of *R. leucaenae* under environmentally stressful conditions.

## Results and discussion

### General characteristics and comparison of *R. leucaenae* genomes

Sequencing of CFN 299^T^ and CPAO 29.8 resulted in high-quality draft genomes with average coverages of 251-fold and 155-fold, respectively. In CFN 299^T^, the genome was assembled in 95 contigs with an N50 size of 296,137 bp, whereas in CPAO 29.8 there are 179 contigs with an N50 size of 219,636 bp.

Both strains of *R. leucaenae* present genomes of similar size and composition. CFN 299^T^ has a 6,694,130-bp and CPAO 29.8 a 6,850,073-bp genome. General features and statistics of the genomes are presented in Table 1. The chromosomes of both strains contain three ribosomal operons. CFN 299^T^ has four plasmids of approximately 1.9 Mb, 500 kb, 220 kb and 190 kb, and apparently the same for CPAO 29.8. Both strains have a megaplasmid and the second largest replicon is the symbiotic plasmid (pSym).

**Table 1:** General statistics of the *Rhizobium leucaenae* strains CFN 299^T^ and CPAO 29.8 genome assemblies and annotations

	CFN 299^T^	CPAO 29.8
Estimated genome size (bp)	6,694,130	6,850,073
N50	296,137	219,636
Size of largest contig	553,642	553,487
Number of contigs	95	179
G + C content (%)	59	59
Coverage	251	155
Number of predicted genes	7,069	6,951
CDS	7,015	6,899
With function	4,664 (66 %)	4,941 (72 %)
Hypothetical	2,351 (34 %)	1,958 (28 %)
tRNA	51	49
rRNA	3	3

Totals of 7015 and 6899 coding DNA sequences (CDS) were predicted for CFN 299^T^ and CPAO 29.8, respectively (Table 1). Putative functions could be assigned to around 66 and 72 % of the CDS from both strains, respectively.

After combining the annotation obtained by RAST and a detailed manual curation process, the information about each contig, gene product and localization in the genomes of CFN 299^T^ and CPAO 29.8 was included in Additional file 1: Table S1. In addition, we made comparisons with other genomes of related *Rhizobium* species, including *R. tropici* CIAT 899^T^, *R. freirei* PRF 81^T^, *R. rhizogenes* K84, *R. etli* CFN 42^T^, *R. phaseoli* CIAT 652, *R. grahanii* CCGE 502^T^, *R. leguminosarum* 3841, *R. mesoamericanum* STM 3625^T^, and other genomes.

Functional classification in COG of the CDS was very similar in both strains, but the two highest categories were of general function and unknown functions (Table 2), highlighting our still poor knowledge about bacterial genomes. Similar percentages of genes with unknown function were revealed by the RAST functional classification system.

**Table 2:** Functional classification in COG of CDSs of *Rhizobium leucaenae* strains CFN 299^T^ and CPAO 29.8

COG functional category	# of CDSs in CFN 299^T^	# of CDSs in CPAO 29.8
C-Energy production and conversion	315	325
D-Cell cycle control, cell division, chromosome partitioning	38	37
E-Amino acid transport and metabolism	471	472
F-Nucleotide transport and metabolism	81	83
G-Carbohydrate transport and metabolism	565	535
H-Coenzyme transport and metabolism	166	170
I-Lipid transport and metabolism	143	141
J-Translation, ribosomal structure and biogenesis	181	182
K-Transcription	467	465
L-Replication, recombination and repair	251	239
M-Cell wall/membrane/envelope biogenesis	232	242
N-Cell motility	58	59
O-Posttranslational modification, protein turnover, chaperones	158	161
P-Inorganic ion transport and metabolism	219	228
Q-Secondary metabolites biosynthesis, transport and catabolism	79	81
R-General function prediction only	679	686
S-Function unknown	538	535
T-Signal transduction mechanisms	201	214
U-Intracellular trafficking, secretion, and vesicular transport	109	118
V-Defense mechanisms	72	74
NO COG	1992	1852

The chromosomes coded for most genes assigned to functionally important classes, predominantly of central metabolism, cellular processes, DNA metabolism, and several CDS related to transport. The great majority of the genes related to stress tolerance that will be commented upon are also in the chromosome, although there were few, but important genes in the pSym. The megaplasmid encoded several hypothetical genes, conjugal transfer *tra* and *trb* operons, and several other classes of genes, including many transporters. pSym encoded all nodulation and symbiosis-related genes, in addition to many hypothetical CDS and transporters (Additional file 1: Table S1).

**Fig. 1:**
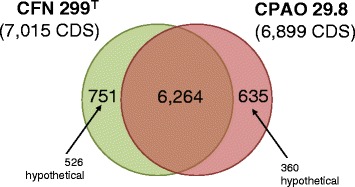
Venn diagram showing the number of orthologous gene clusters shared by *Rhizobium leucaenae* strains CFN 299^T^ and CPAO 29.8. Based on RAST predicted genes and manual curation

**Fig. 2:**
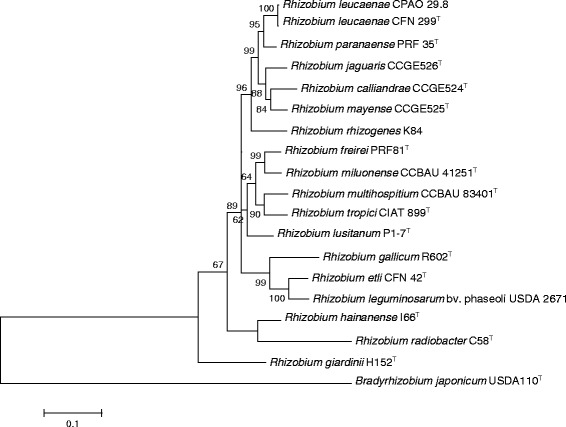
Neighbor joining phylogenetic tree based on a concatenated alignment of *recA, glnII* and *gyrB* sequences of *Rhizobium leucaenae* strains from this study and other type/reference strains. Bootstrap support values 70 % or greater are shown at tree nodes

**Fig. 3:**
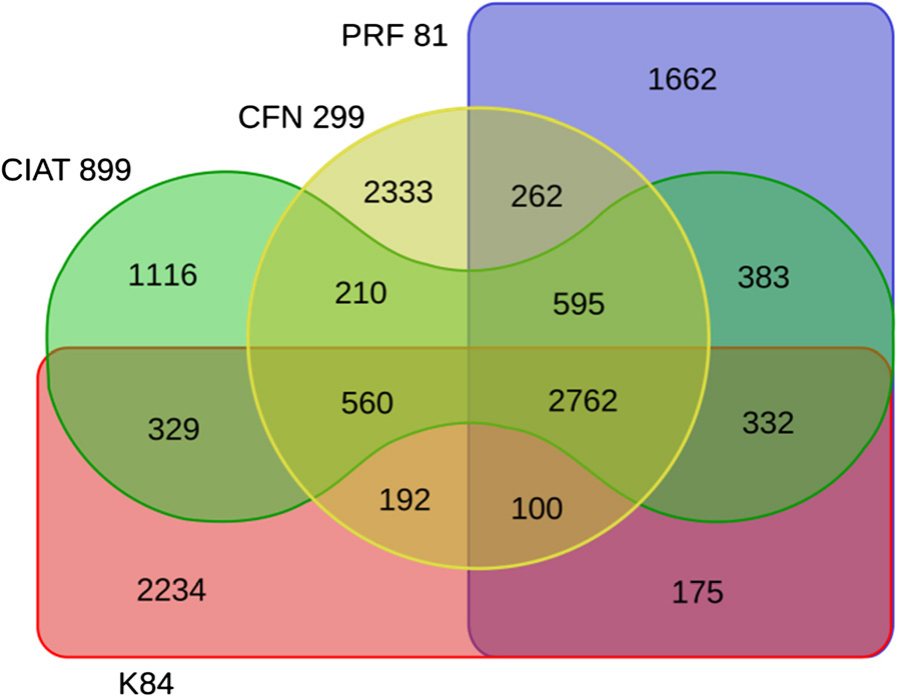
Venn diagram showing the number of orthologous gene clusters shared by *Rhizobium leucaenae* strains CFN 299^T^ and *Rhizobium tropici* CIAT 899^T^, *Rhizobium freirei* PRF 81^T^ and *Rhizobium rhizogenes* K84. Based on RAST predicted genes and manual curation

Genome comparisons indicated high similarities between CFN 299^T^ and CPAO 29.8 strains, with 6264 CDS in common (Fig. 1). CFN 299^T^ presented 751 unique CDS, 70 % of which with hypothetical functions, while CPAO 29.8 presented 635 unique CDS, 57 % with hypothetical functions. Genes unique to CFN 299^T^ included conjugation genes and sugar transporters and catabolic genes, and several of these genes were located in the smallest plasmid of this strain. On the other hand, CPAO 29.8 specific loci included a prophage, a cluster of flagellum-related genes, conjugation genes, and carbohydrate transporters. The phylogeny of this interesting group of strains classified in the “*R. tropici* group” is shown in Fig. 2, and considering the comparison of CFN 299^T^ with *R. tropici* CIAT 899^T^, *R. freirei* PRF 81^T^ and *R. rhizogenes* K84, we found that CFN 299^T^ carries a high number of exclusive CDS—2333 (Fig. 3).

### Searching for genes related to the high tolerance of environmental stress in *R. leucaenae*

One major limiting factor in agriculture results from edaphoclimatic stressful conditions, in the tropics represented by high temperatures, acidic soils, salinity and water deficiency, all affecting both the host plant and the bacterium; the symbiosis of common bean-rhizobium seems particularly sensitive to environmental stresses [16, 17]. Both CFN 299^T^ and CPAO 29.8 are capable of tolerating environmental stresses, including high temperatures (37 °C) and acidity (pH 4.0) [6, 9], and the species apparently encompasses the great majority of strains isolated from nodules on common bean and leucaena in the Cerrados region [6, 10, 18], an edaphic type of savannah covering 207 million hectares of Brazilian land (25 %) that are frequently exposed to harsh environmental stressful conditions. Therefore, investigating genes related to the stress-tolerance features of *R. leucaenae* may not only help to understand the successful saprophytic and symbiotic strategies used by the species, but also contribute to the identification of genes with biotechnological potential. In Additional file 2: Table S2, we included the genes related to stress tolerance in both strains, and below we discuss the main categories.

### pH stress

Besides the need to survive in acid soils, rhizobial strains may face pH stresses in the process of establishment of the symbioses, and also when the bacterium is inside the symbiosome; in both situations, the challenge is to maintain the intracellular pH around 7.2 to 7.5 [19]. To ensure intracellular pH homeostasis when submitted to acid conditions, bacteria may take advantage of different mechanisms. One major strategy consists of proton transport mediated by proton pumps, proton-coupled ATPases and cation-proton antiporters. CFN 299^T^ and CPAO 29.8 genomes present a set of genes—*phaA*, *phaB*, *phaC*, *phaD*, *phaE*, *phaF* and *phaG*—related to different Na^+^/H^+^ antiporter subunits. This antiporter system has been described in cell defenses in alkaline conditions, in which intracellular H^+^ is replaced by Na^+^ [19, 20]. The accumulation of positively charged ions, such as Na^+^ and K^+^, added to the efflux of H^+^ also seems important when cells are exposed to acid stress [21].

Glutathione synthase (*gshB*) is involved in acid tolerance of *R. tropici* CIAT 899^T^, since an auxotrophic mutant showed reduced growth at pH 5.0, with the suggestion that it could be related to a lower concentration of intracellular K^+^ compared to the wild-type strain [22]. The same authors suggested that the low intracellular K^+^ content may be related to the high activity of the KefB/KefC glutathione-regulator K^+^ efflux transporter verified in the absence of glutathione [22]. Muglia et al. [23] also emphasized the importance of glutathione to the acid tolerance of *R. tropici* CIAT 899^T^, once the transcription of *gshB* is activated at low pH. Both glutathione synthase and K^+^-efflux transporter genes *ghsB* and *kefB* are present in the genomes of CFN 299^T^ and CPAO 29.8.

As in *R. tropici* CIAT 899^T^ and *R. freirei* PRF 81^T^ genomes [11], *R. leucaenae* stains CPAO 29.8 and CFN 299^T^ possess two copies of the gene *cfa,* encoding a cyclopropane-fatty-acyl-phospholipid synthase protein. In *Escherichia coli*, this gene converts unsaturated fatty acids to saturated counterparts, modifying the inner-membrane phospholipids, and leading to an acid-tolerant phenotype [24]. One possible mechanism would be that the changes in the inner membrane reduce the permeability to H^+^ [25]. Genes coding for ClC-type chloride channels have also been reported as playing a role in acid-pH tolerance by controlling the intracellular H^+^ concentration. In a low-pH environment, chloride channels accomplish proton extrusion, maintaining the cell homeostasis [26]. Three genes of the widespread ClC-family are present in the chromosomes of CPAO 29.8 and CFN 299^T^. These paralogous genes may present different functions in addition to their protective roles in acid-pH stress, since the knockout of *sycA,* a gene from the ClC-family, results in a symbiotically defective phenotype [27].

The cellular membrane is a crucial component for avoidance of pH stress, and tolerance may be related to structural modifications, either by changes in the lipid composition, or by allocating several proteins involved in transport of compounds to maintain the intracellular pH homeostasis. It is equally true that some genes involved in membrane composition and with a role in pH-stress defense can also contribute to the success of the symbiosis. Thus, when CIAT 899^T^ is submitted to low pH conditions, an increase of ornithine lipids (OL) and their hydroxylation are observed in the outer membrane [28]. The gene coding for ornithine lipid biosynthesis, *olsC*, is present in the chromosomes of CFN 299^T^ and CPAO 29.8 and, according to Rojas-Jiménez et al. [27], is responsible for the OL biosynthesis and for the addition of the hydroxyl group, suggesting a protective role to acid stress. Indeed, a CIAT 899^T^*olsC* mutant showed significant growth reduction at pH 4.5, as well as generated symbiotic defects, resulting in poor development of the nodules and a two-fold reduction in nitrogen fixation [28].

In CIAT 899^T^, the genes *lipA* and *atvA* were up-regulated when the bacterium was exposed to an experimental acid condition [29]. Among the CFN 299^T^ and CPAO 29.8 genes, we found *lipA*, which is required for lysyl-phosphatidylglycerol biosynthesis, showing a relation with antimicrobial resistance and competitiveness [30], while *atvA* participates in the membrane lipids metabolism [29], both studied in CIAT 899^T^. Complex pleiotropic phenotypes were observed in CIAT 899^T^*atvA* auxotroph, including acid sensitivity [30]. However, the acid-sensitive mutants were not affected in competitiveness or nitrogen fixation capacity, but in contrast, the *lipA* CIAT 899^T^ mutant exhibited a seven-fold reduction in competitiveness of nodulation in comparison to the wild type [29].

### High-temperature stress

Although the optimum growth temperature for most rhizobia ranges from 25 to 30 °C, outlier temperatures are often experienced at the rhizosphere, affecting both growth and saprophytic competence. Besides affecting survival in the free-living state, heat stress negatively affects molecular-signal exchange, root infection, nodulation and several steps of the nitrogen fixation process [9, 16, 17, 31], and one major feature of *R. leucaenae* and *R. tropici* is their superior symbiotic performance under high temperature conditions in comparison to species that do not belong to the “*R. tropici* group” (Additional file 3: Table S3).

The ability of *R. leucaenae* to grow at high temperature (37 °C) [6] might be related to several genes that were identified in CFN 299^T^ and CPAO 29.8 genomes. In the closely related species *R. freirei* PRF 81^T^, Gomes et al. [32] identified 54 proteins in response to heat stress, 38 of which were now recognized in *R. leucaenae*. This set of genes comprises the molecular bases of temperature-stress responses, being responsible for rapid physiological changes, and many of them are under the transcription control of the RpoH alternative sigma factor [33].

Heat-shock proteins (HSP) play key roles in repair of heat-stress damages. These proteins are distributed in different groups, including the major chaperone-system proteins, the small heat-shock proteins and the chaperone-like proteins, all of which are represented by clusters of genes in both of the CFN 299^T^ and CPAO 29.8 genomes. The major chaperones, DnaK, DnaJ and GrpE, compose the DnaK system, which is the most versatile chaperone system, responsible for *de novo* protein folding, protein transport and for the increase on RpoH stability during heat stress, indirectly assisting the gene expression aimed at cell defense against high temperatures. In complement, the GroEL system, comprising the GroES protein, can routinely rescue more than 80 % of the proteins damaged by heat [33, 34].

The small heat-shock proteins (sHSP), as their denomination indicates, present low molecular mass and are involved in reversing protein aggregations generated under high temperatures, keeping them in a folding-competent state [35]. A cluster of small HSPs genes is harbored in the chromosomes of the *R. leucaenae* genomes, especially those correlated with the HSP20 family, among them, *ibpA, hspG and hspH*, *hspH*; the last of which is up-regulated in *Bradyrhizobium japonicum* under heat-stress conditions [36]. In *E. coli*, for example, only two of these sHSP genes are presen, but different from most bacteria, the occurrence of several genes encoding these small proteins seem to be typical of rhizobial species [11, 35]. In another study, the comparison of heat-tolerant and -sensitive *Rhizobium* strains revealed the overexpression of different sHSP by the tolerant strain in response to increased temperature [37]. The same is expected for CFN 299^T^ and CPAO 29.8 strains, once they are capable of growing at temperatures above the optimum conditions for most rhizobial species. Curiously, two genes of HSP20 are located at the CFN 299^T^ and CPAO 29.8 symbiotic plasmids, as well as in CIAT 899^T^ and PRF 81^T^, suggesting a probable role in cell defense during the symbiosis [11].

Apart from the classical chaperones, other proteins can display a heat-stress-protective role in addition to their main function. Within this group of chaperone-like proteins are the translation factors EF-Tu, EF-G and IF2, which help in protein folding and prevent unfolding proteins from forming aggregates during high-temperature conditions [38, 39]. The genes that encode these chaperone-like proteins are up-regulated in PRF 81^T^ at 37 °C [32]. Gene-deletion studies have improved understanding of how the cell endures the heat stress by revealing new genes that are indirectly associated with high-temperature tolerance, but confer tolerant phenotypes [31]. For example, *R. etli relA* and *R. tropici guaB* mutants had their ability to tolerate high temperatures decreased [40]. Both *relA* and *guaB*, in addition to the genes coding for translation factors, EF-Tu, EF-G and IF2, which were up-regulated in PRF 81^T^, are present in the CFN 299^T^ and CPAO 29.8 genomes; therefore, they might be related to the heat tolerance of this strain.

### Osmotic stress

Osmotic stress can affect bacteria by loss of intracellular water or by excessive water influx, depending on the environmental conditions, and soil desiccation and salinity are the main factors related to osmotic stress. Salinity, for example, affects almost 40 % of land globally, potentially negatively impacting soil bacteria [2, 41]. In rhizobia, desiccation and salinity also affect the nitrogen-fixation process [41]. To overcome osmotic stress, the cells have protective mechanisms and, in the genomes of *R. leucaena*e strains CPAO 29.8 and CFN 299^T^, we found more than 30 genes related to osmotolerance.

The initial response of *Rhizobium* strains to cope with osmotic stress relies on the uptake and accumulation of potassium (K^+^) [42]. A high-affinity K^+^ (Kup) system was previously reported in *R. tropici* and *R. freirei* [11, 43] and the gene is present in the chromosomes of CFN 299^T^ and CPAO 29.8. After uptake, the K^+^ ion acts as a secondary messenger driving other responses to osmotic stress, and in CFN 299^T^ and in CPAO 29.8, we found genes encoding the KdpABC K^+^ transporting ATPase, also present in strains CIAT 899^T^ and PRF 81^T^ [11].

Another common strategy for coping with hyperosmotic environments is the synthesis and accumulation of compatible solutes, including trehalose, glycine, betaine, proline and ectoine [44]. Such compounds are called compatible because they are not harmful to macromolecules and their diversity is important for bacterial adaptation to environmental changes, which involve the amount of water and, in the rhizosphere, the salts and other exudates from plants [45]. Trehalose, for example, was described as an osmoprotectant in *Sinorhizobium meliloti* [46], besides being involved in protection against other abiotic stresses [44]. It is also implicated in the nodulation process of *S. meliloti* [43], *B. japonicum* [47], *R. etli* [48] and *R. leguminosarum* bv. *trifolii* [49]. *R. leucaenae* presents the genes *otsA* and *otsB* that encode enzymes involved in trehalose biosynthesis from UDP-glucose and glucose 6-phosphate [49]. The additional pathway that involves trehalose synthase (TreS), an enzyme present in CIAT 899^T^ that catalyzes trehalose synthesis from maltose [50], is also present in the chromosomes of CFN 299^T^ and CPAO 29.8.

Among the osmoprotectants, glycine betaine can be taken up or synthesized from choline [51]. The presence of this compatible solute in the medium improves the growth rate of *R. tropici*, *S. meliloti*, *S. fredii* and *R. galegae* when submitted to 300 mM of NaCl [51]. In such conditions, the enzyme activity for glycine betaine degradation decreases, while the enzymes’ activities converting choline to glycine betaine— choline dehydrogenase (BetA) and betaine aldehyde dehydrogenase (BetB)—increase [51]. The external choline could be taken by the ChoXWV ABC-type transporters [52], also encoded in *R. leucaenae* strains CPAO 29.8 and CFN 299^T^. Genes *betA* and *betB* are also present in the genomes of both strains of *R. leucaenae*, as well as *betC*, which encodes choline sulfatase and may use choline sulfate as a precursor to glycine betaine synthesis [11]. The uptake of proline betaine performed by the transporters genes—*prbA*, *prbB*, *prbC* and *prbD*—can also overcome the osmotic stress.

Contrasting with hyper-osmotic stress, less is known of how bacteria cope with hypo-osmotic shock. Dylan et al. [53] reported that a low-osmolarity medium leads to a striking increase in the level of β-(1 → 2)-glucans in *S. meliloti* periplasm space. This finding is directly related with the *ndv* locus, which includes two genes, *ndvA* and *ndvB* [54], both harbored in the chromosome of strains CFN 299^T^ and CPAO 29.8. NdvB and NdvA are responsible for the synthesis of cyclic beta glucans (CbG) and their translocation to the periplasmic space, roles that are essential for nodulation [54]. Mutants in *ndvA* and *ndvB* genes implied in the absence of CbG, generate disturbances associated with hypo-osmotic adaptation, motility, root attachment and infection [53]. Increases in the membrane turgor pressure, caused by the movement of water into the cell cytoplasm in a low-osmotic environment, induce the activation of mechanosensitive (MS) channels [54, 55]. There are two major classes of bacterial MS channels, MscS and MscL, which, under hypo-osmotic conditions, perform the release of internal solutes to equilibrate intra and extracellular osmolarity [55]. Whereas the MscL channel activity is usually the product of a single gene, many bacteria possess multiple MscS homologues, including *R. leucaenae* CFN 299^T^ and CPAO 29.8, as well as *R. tropici* CIAT 899^T^, which presents five genes encoding MscS. Multiple genes for MS channels may offer the cell higher tolerance to hypo-osmotic shocks.

Changes in the osmolarity activate fluxes of solutes and water along the concentration gradient. In addition to solutes, water flows through the membranes of living cells by two distinct mechanisms, i.e. by simple diffusion and by water-selective channel proteins, namely aquaporins [56]. Widespread in a variety of organisms, the occurrence of aquaporins in bacteria suggests that they could be involved in osmo-adaptation responses, which was confirmed in *E. coli* under hypo-osmotic conditions [57] and in *Brucella abortus* exposed to a hypertonic environment [56]. As observed in *R. tropici* CIAT 899^T^ [11], DNA sequences for two aquaporins were identified in the genomes of the CFN 299^T^ and CPAO 29.8, one in the chromosome and the other in the symbiotic plasmid.

### Oxidative stress

Aerobic bacteria, including rhizobia, have to deal with reactive oxygen species (ROS) such as the superoxide anion (O_2_^−^) and hydrogen peroxide (H_2_O_2_), which are produced mainly by cellular respiration [58]. To avoid the detrimental effects generated by oxidative stress agents, *R. leucaenae* strains CPAO 29.8 and CFN 299^T^ possess a cluster of genes the products of which are related to the detoxification of ROS. Additionally, rhizobia are exposed to oxidant agents in the rhizosphere when in the free-living stage, during the infection process [59] and inside the nodules [60]. This reflects the importance of tolerating and overcoming oxidative stress as one essential feature for achieving an effective symbiosis.

Different mechanisms of protection against the ROS have been described, including catalases, superoxide dismutases (SODs), peroxidases and other enzymes such as peroxiredoxin [59–61]. The oxidative-stress responses to superoxide compounds are induced by the SoxR transcriptional activator [11], leading to the expression of genes for superoxide dismutase. Together with *soxR,* two genes for SOD, *sodC* and *sodM*, are harbored in the genomes of CFN 299^T^ and CPAO 29.8, as well as in CIAT 899^T^ and *R. freirei* PRF 81^T^ [11]. Regarded as the most important factor in protection and maintenance of oxidative homeostasis in bacteria, SOD catalyzes the dismutation of O_2_^−^ to H_2_O_2_ and O_2_ [59]. The responses to H_2_O_2_, in turn, are driven by the OxyR transcription factor [62], a key regulator encoded by the CPAO 29.8 and CFN 299^T^ genomes. Responses to oxidative stress include also the expression of catalases, which break down H_2_O_2_ to oxygen and water [61, 63]. Two genes encoding catalases were found in the genomes of *R. leucaenae*, *katG* for a bifunctional catalase-peroxidase (HPI) [64] and another encoding a monofuntional catalase. The expression of catalases is crucial during plant infection [65], because hydrogen peroxide is one of the plant defenses experienced by symbiotic microorganisms [66]. In this adverse environment, rhizobia have to tolerate the oxidative stress, but should also maintain a correct balance of H_2_O_2_ for the establishment of a successful symbiosis [61]. This was demonstrated by over-expressing the *katB* gene of *S. meliloti*, inducing a lower number of nodules on *M. sativa*, probably reflecting the intense reduction of H_2_O_2_ in comparison to the wild-type strain [61].

Organic hydroperoxides (OHRs) are highly harmful to cells and, and together with H_2_O_2_, represent an important component of plant defense during bacterial infection [67]. Organic hydroperoxide resistance (*ohr*) proteins are related to hydroperoxide detoxification, and as in PRF 81^T^ [11], CFN 299^T^ and CPAO 29.8 present three *ohr* copies, in addition to their putative organic peroxide-inducible transcription repressor (*ohrR*) gene. Resistance to organic hydroperoxides in *S. meliloti* requires *ohr* and *ohrR* gene expression; however, their gene products are not essential for overcoming the oxidative condition faced during the nodulation, since *ohr* and *ohrR* auxotroph mutants form effective nodules [67]. It has been suggested that the alkyl hydroperoxidase reductase protein detoxifies the plant’s OHRs during the symbiosis establishment [67]. Two genes encoding putative alkyl hydroperoxidase reductase proteins, members of the peroxiredoxin family, were detected in CFN 299^T^ and CPAO 29.8. Such peroxiredoxines represent the main enzymes for the detoxification of organic peroxides, reducing them to alcohols [68]. Another peroxiredoxin gene (*prxS*) was found in the symbiotic plasmid of the two *R. leucaenae* strains. Its induction has already been reported in rhizobial bacteroids, detected both by transcriptomics and proteomics [63, 69], suggesting the involvement of this gene product with ROS detoxification during the nitrogen-fixation process [60].

As explained, bacteria possess several mechanisms to avoid damage caused by oxidative stresses. However, if ROS are not efficiently detoxified, it is necessary that the damaged molecules return to their original redox states. Methionine sulfoxide reductase activity (Msr) may repair damaged proteins during oxidative stress [70]. *msrA* and *msrB* genes, present in two copies on the CFN 299^T^ and CPAO 29.8 genomes, encode Msr proteins that provide the reversion of methionine from its oxidized to the reduced state [71]; they were also detected in *R. tropici* and *R. freirei* genomes [11].

### Secretion systems

Protein secretion by the type-I secretion system (T1SS), a Sec-independent system to export proteins (usually proteases) from Gram-negative bacteria, occurs through an oligomeric protein channel composed of an inner membrane ATP-binding cassette (ABC) protein, a periplasmic membrane fusion protein (MFP, the HylD protein), and a pore-forming outer-membrane protein (OMP) [72]. Ten copies of the *hylD* genes were found in the CFN 299^T^ and CPAO 29.8 genomes. However, a single copy of the TolC OMP was found in these genomes, close to a cluster that included HlyD and one ATPase. In the case of CFN 299^T^, this region was close to a gene that encodes an RTX protein (repeats in toxin), that is secreted by the T1SS [73]. This protein is not present in the genome of CPAO 29.8. The CFN 299^T^ RTX protein contains nine T1SS_rpt_143 domains and the typical carboxy-terminal, glycine- and aspartate-rich repeats.

Proteins secreted by the T2SS depend on the Sec or Tat systems for initial transport into the periplasm. Once there, they pass through the outer membrane via a multimeric (12–14 subunits) complex of pore-forming secreting proteins. Both genomes present the TadBCDE proteins with 100 % identity to each other, and 91, 94, 89 and 85 % identical, respectively, to these proteins in CIAT 899^T^. The *tad* genes (from *t*igh *ad*herence) encode functions necessary for the biogenesis of the Flp subfamily of type IVb [74].

Type-IV pili are virulence factors in various bacteria and mediate, among other functions, the colonization of surfaces in different genera of Gram-negative bacteria, including *Haemophilus*, *Pasteurella*, *Pseudomonas* and *Yersinia* [75]. The *tad*-like genes found in the genome of *Micrococcus luteus* are also required for genetic transformation in this actinobacterial species [76]. Both strains of *R. leucaenae* harbor an identical *cpaABCDEF* cluster, which encodes the collagen-binding pilus component. The genes that encode these proteins are also present in the genome of CIAT 899^T^, with identities of 69, 82, 90, 73, 86 and 91 %, respectively.

Type-IV secretion systems (T4SS) are able to transfer proteins or nucleoprotein complexes across membranes [77]. We found T4SSs in the CFN 299^T^ and CPAO 29.8 genomes. In both strains we found *virD4* and *virB1*/*virB11* genes that are not present in the CIAT 899^T^genome, but *virD4* is present in the PRF 81^T^ genome. *virD4* and *virB3*-*B11* of *R. leucaenae* are located in the same cluster, whereas *virB1virB2* and *virb7*-like are located in separate regions. The *vir* system of *Agrobacterium tumefaciens* is responsible for the transference of tumorigenic DNA (T-DNA) into plant cells [78], while in *Mesorhizobium loti* R7A, a VirB/VirD4 system acts in the translocation of effector proteins into host cells, affecting the symbiosis in a host-dependant manner [79]. In *R. leucaenae*, we did not find homologues to the two-component regulatory system VirA/VirG that controls expression of *virB*/*virD4* systems in *A. tumefaciens* and *M. loti*. However, we found in both strains three regions containing the F-type *tra*/*trb* T4SS genes, required for conjugal transfer of plasmids. The first of these regions, located in pA, contained the cluster *traGDCAFBH*, adjacent to the cluster *traMRtrbHIGFLJEDCBtraI*. However, in CPAO 29.8, the genes *traRtrbHIGFLJE* and *traI* were not present*,* suggesting that the conjugation system may not be functional. This region is similar to the conjugation machineries present in *R. etli* CFN 42^T^ and Mim1, *R. tropici* CIAT 899^T^ and *S. fredii* GR64. The second region contains *traGDCAFBH* close to *trbIHGFLKJEDCB*, and only *trbD* is not present in CPAO 29.8. The *tra* genes of this second region are similar to those present in *S. meliloti* 41, *S. fredii* NGR234, and *A. tumefaciens* plasmid pRiA4b; the *trb* region is similar to that found in *R. etli* CFN 42^T^, *S. fredii* NGR234 and *S. meliloti* 41. The third region is located in the pSym and contains the genes *traGDCAFBHMRtrbIHGFLJEDCBtraI*, and only the conjugational transfer transcriptional repressor TraR is not present in CPAO 29.8, which may represent a constitutive repression of the conjugation system in this strain. This pSym region is highly homologous to the one in the pSym of *R. tropici* CIAT 899^T^ and also showed high homology with regions present in *R. etli* CFN 42^T^ and *S. fredii* GR4. The presence of *traI* suggests that the transcription of these genes is regulated by quorum-sensing mechanisms involving N-acyl homoserine lactones. In the pSym of CIAT 899^T^, an IS256 inserted upstream of *traR* may have disrupted its promoter, leading to a constitutive repression of the conjugation system of this plasmid. However, this transposase is not present in the CFN 299^T^ pSym and the region is not constitutively repressed. All three F-type T4SSs identified in the CFN 299^T^ and CPAO 29.8 genomes were adjacent to *repABC* genes. However, in the first T4SS region of CPAO 29.8, we have not found *repC*, although we still have a draft genome.

The type-V secretion system (T5SS) allows secretion of large proteins that act as virulence factors [80]. In the genomes of CFN 299^T^ and CPAO 29.8, a channel-forming transporter/cytolysins activator of the TpsB family was found, but not the TpsA protein that encodes a filamentous hemagglutinin-like T5SS-secreted protein. TpsBs are 100 % identical to each other in *R. leucaenae* and 79 % to *R. tropici* CIAT 899^T^, and form a beta-barrel domain for T5SS-secretion proteins. However, only one auto-transporter, a pertactin-like passenger domain (virulence factors), C-terminal, subgroup 2, is present in the genome of CFN 299^T^ but not in CPAO 29.8.

In conclusion, both genomes of *R. leucaenae* are rich in CDS for genes of secretion systems. Type I, II and V can help in the exportation/secretion of proteins, and might be implied in giving competitive ability to the bacteria, especially under stressful conditions. Type-IV pili can help in colonization and, therefore, lead to a successful competitive ability in relation to other soil microorganisms, facilitating root infection and nodulation. Type-IV genes are particularly interesting for their role as effector proteins in host cells. It is interesting that such effectors were found to be expressed at high temperatures in *R. freirei* PRF 81^T^ [32], enabling tolerance of stresses, and better establishment of the symbiosis under such conditions deserves fuller investigation.

### Symbiotic features

#### The symbiotic plasmid

**Fig. 4:**
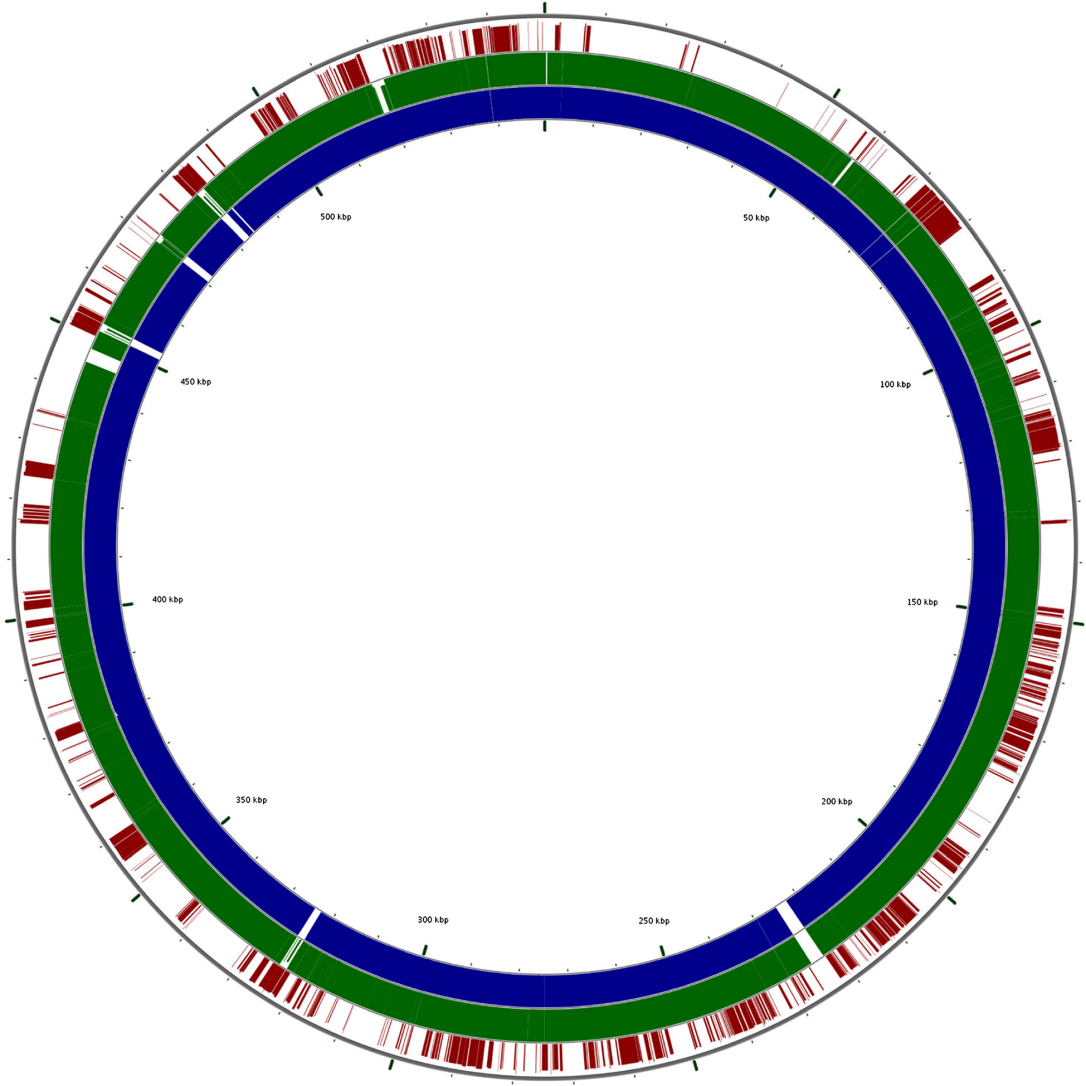
Conservation between the symbiotic plasmids of *R. tropici* CIAT 899^T^, *R. leucaenae* CFN 299^T^, *R. leucaenae* CPAO 29.8 and *R. gallicum* R602^T^, reinforcing the suggestion of a common symbiotic plasmid defining the symbiovar tropici. *Circles* from innermost to outermost depict BLASTN matches between CIAT 899 and CFN 299 (*blue*), CPAO 29.8 (*green*) or R602 (*red*)

It is worth remembering that legume nodulation requires a cascade of molecular signals exchanged between the host plant and the rhizobium. This molecular dialogue begins with the exudation of molecules—mostly flavonoids—from the legume, which are recognized by the bacterium. When induced by the plant-host molecules, rhizobia synthesize lipochitooligosaccharides (LCOs), also known as Nod factors, responsible for launching the nodulation process, and the whole process is orchestrated by a set of nodulation (*nod*) genes [81]. In *Rhizobium*, *nod* genes are present in the symbiotic plasmid, which, in *R. leucaenae* CFN 299^T^ and CPAO 29.8, showed high similarity (>99 %) with the pSym of *R. tropici* CIAT 899^T^. This finding gives strength to the suggestion of a conserved symbiotic plasmid defining the symbiovar tropici of species belonging to the *R. tropici* “group” [11]. Figure 4 displays the conservation between the pSym of *R. tropici* CIAT 899^T^, *R. leucaenae* CFN 299^T^ and *R. leucaenae* CPAO 29.8.

Comparisons of similarities of each *nod* gene of *R. leucaenae* CFN 299^T^ with those of strains CPAO 29.8 and *R. tropici* CIAT 899^T^ are shown in Additional file 4: Table S4. Few differences were detected, and included full similarity but slightly lower coverage, of *nodA1* and *nodA3* genes, and apparently CFN 299^T^ carries both a truncated and a full copy of the *nodS* gene. The synthesis of the Nod factor-backbone chitin oligosaccharide structure is driven by *nodC,* and *nodS* is one of the genes responsible for the decoration of the basic structure, the methylation, an important property to define host range [82]. However, possible evolutionary events and biological implications of an additional truncated *nodS* are still to be determined.

Interestingly, when *nod* genes of *R. leucaenae* CPAO 29.8 were compared with CFN 299^T^ and *R. tropici* CIAT 899^T^, a difference was detected in *nodD3,* with full identity but lower coverage than the other homologues (Additional file 5: Table S5). Analysing the genomic region, we verified that this occurred due to the presence of a mobile element, that in CIAT 899^T^ corresponds to one putative IS21 family transposase *OrfA* y *OrfB*” (>90 % identity), that has been interposed inside the gene. Recently, *nodD1* of CIAT 899^T^ was recognized as the most important regulatory *nodD* gene for nodulation of common bean and leucaena [13], and *nodD3* was shown to be an activator of *nodD1* [12]. However, in CPAO 29.8, apparently the transposon is not affecting the symbiotic performance, as the strain is highly effective in nodulating and fixing nitrogen with common bean (Additional file 3: Table S3) and also with leucaena (data not shown).

### Synthesis of Nod factors under abiotic stress

The most important agronomic feature of the two *R. leucaenae* strains from our study is their high capacity of nodulating and fixing nitrogen with common bean even under stressful environmental conditions. These properties might be at least partially related to the capacity of producing Nod factors under adverse conditions, as observed in *R. tropici* CIAT 899^T^ under saline stress [12–15]. We have recently raised the hypothesis that the release of a large set of Nod factors by *R. tropici* CIAT 899^T^ under saline stress and in the absence of plant signals might represent a strategy: *i)* to nodulate a broad range of hosts under stressful conditions, as an evolutionary strategy to perpetuate the symbiosis; *ii)* to confer stress tolerance to the bacterium, in a mechanism not yet elucidated [12, 13].

To get a better understanding of the biosynthesis of Nod factors under abiotic stress, we made comparisons with other rhizobial strains symbionts of common bean. TLC chromatographic profiles of Nod factors in the presence and absence of flavonoids and abiotic stresses were obtained for *R. leucaenae* CFN 299^T^, *R. tropici* CIAT 899^T^, *R. freirei* PRF 81^T^, *R. leguminosarum* bv *phaseoli* strain TAL1121, *R. etli* strains CFN 42^T^, Sc15, ISp19 and ISP36, *R. giardinii* H152^T^ and *R. gallicum* R602^T^. None of the studied strains synthesized Nod factors in the absence of the *nod*-gene inducer apigenin when grown at high temperature (37 °C), but it is noteworthy that, in comparison to growth at 28 °C, heat also decreased drastically Nod-factor synthesis even when induced by flavonoids (data not shown). Indeed, nodulation of common bean is highly affected by heat stress [16, 17], and our results indicate that inhibition may start from the release of Nod factors.

**Fig. 5:**
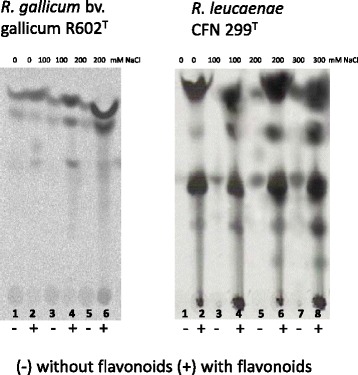
TLC analysis obtained with ^14^C labeled *N*-acetylglucosamine of Nod factors produced by *R. gallicum* bv. gallicum R602^T^ *and R. leucaenae* CFN 299^T^ grown under saline stress. Bacteria were induced (+) or not (−) with a flavonoid nod-gene inducer (apigenin, 3.7 μM), under different levels of saline stress (concentration of NaCl). Lines 1 and 2 represent the control without saline stress

Under acid stress (pH 5.0), although substantially reduced, *R. leucaenae* CFN 299^T^, *R. tropici* CIAT 899^T^, *R. leguminosarum* bv *phaseoli* TAL1121, *R. etli* CFN 42^T^, and *R. gallicum* strains R602^T^ were able to synthesize Nod factors in the absence of flavonoids, but not *R. giardinii* H152^T^, but none was able to synthesize Nod factors under alkaline stress (Additional file 6: Figure S1). However, under osmotic stress (saline) only CFN 299^T^, CIAT 899^T^ and PFR 81^T^, all belonging to the “*R. tropici* group” and R602^T^ synthesized Nod factors in the absence of flavonoids. Figure 5 displays a comparison of Nod-factor profiles in CFN 299^T^ and R602^T^ induced by different concentrations of NaCl.

**Fig. 6:**
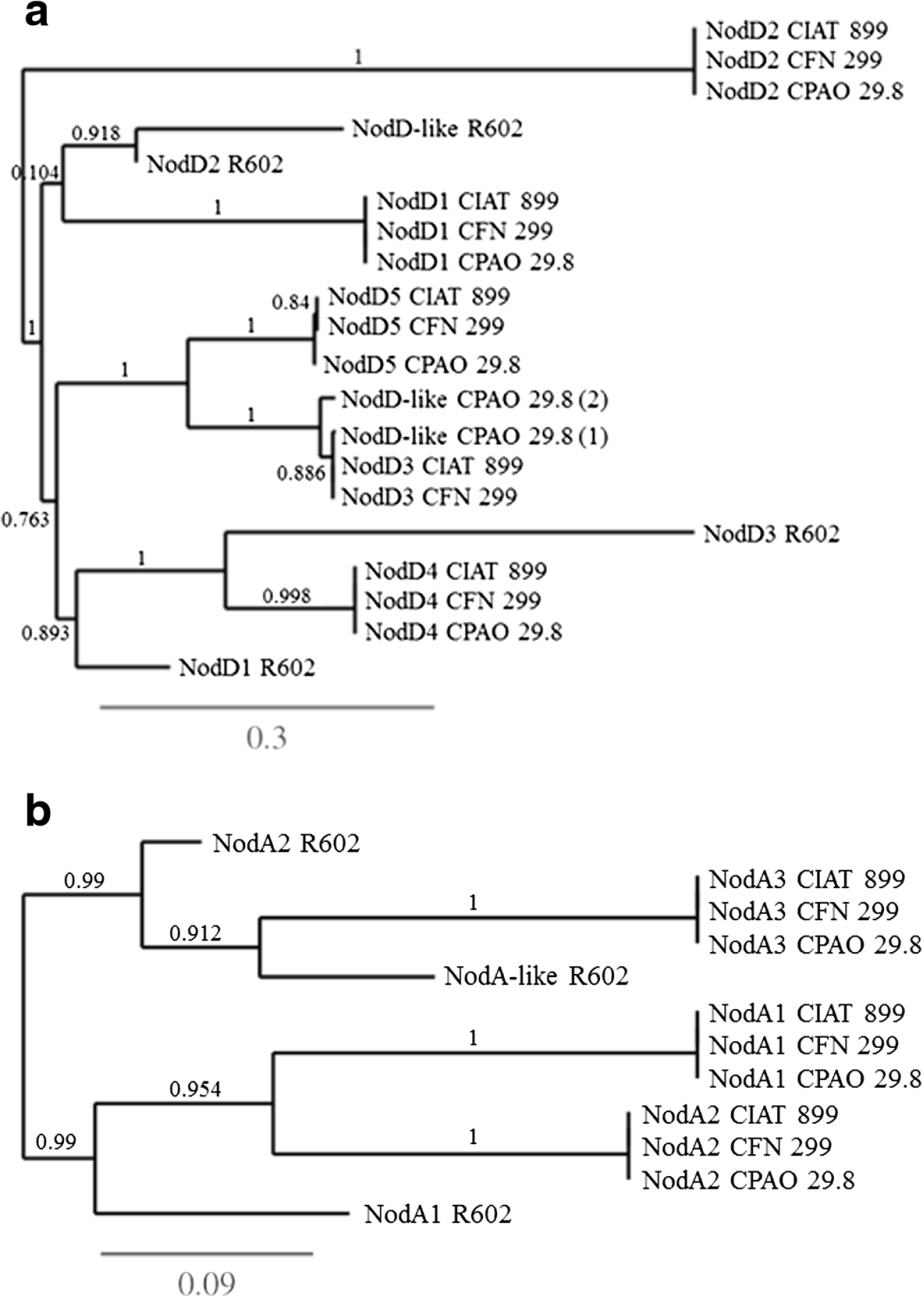
**a** Phylogenetic tree of representatives *nodD* genes of *R. leucaenae* CFN 299^T^ and CPAO 29.8, *R. tropici* CIAT 899^T^ and *R. gallicum* R602^T^. The branches length represents the evolutionary lineages changing over time. The length of the brach represents the amount of changes and it is proportional to the number of nucleotide substitutions per site. The bar at the bottom of the figure provides a scale for the evolution. **b** Phylogenetic tree of the three copies of *nodA* genes of *R. leucaenae* CFN 299^T^ and CPAO 29.8, *R. tropici* CIAT 899^T^ and the two *nodA* copies and a third *nodA*-like of *R. gallicum* R602^T^. The branches length represents the evolutionary lineages changing over time. The length of the brach represents the amount of changes and it is proportional to the number of nucleotide substitutions per site. The bar at the bottom of the figure provides a scale for the evolution

In view of the results of synthesis of Nod factors under saline stress, we concentrated the comparison of nodulation genes on the rhizobial species carrying the pSym-tropici, including the two strains from our study, and also *R. gallicum* bv. gallicum R602^T^, because this was the only species not belonging to the “*R. tropici*” group able to synthesize Nod factors under saline conditions and in the absence of flavonoids. The homology of the pSym-tropici with R602^T^ was of about 92 %. In relation to the main regulatory *nodD* genes, in the pSym of *R. tropici* CIAT 899^T^, *R. leucaenae* CFN 299^T^ and CPAO 29.8 there were five *nodD* copies showing 100 % of homology. *R. gallicum* R602^T^ carries four copies of *nodD* genes, considerably different from the pSym-tropici (Fig. 6a). From recent studies performed with mutants of the five *nodD* genes of *R. tropici*, it has been determined that although all *nodD* copies play a role in the synthesis of Nod factors under abiotic stress, *nodD2* could be a major protagonist [12, 13]. However, none of the *nodD* genes of *R. gallicum* R602^T^ showed high similarity with *nodD2* of *R. tropici*. Another gene that might participate in the *nod*-gene induction by abiotic stress is *nodA*, but, again, in R602^T^ the similarity of the two copies and a third *nodA*-like copy with *nodA* genes of *R. tropici* CIAT 899^T^, CFN 299^T^, and CPAO29.3 (identical to each other) was lower (Fig. 6b). Altogether, these comparisons seem to indicate that, in *R. gallicum* R602^T^, the regulatory induction of Nod-factor synthesis by abiotic stress might be different from that in *R. tropici*.

## Conclusions

A detailed study of the genes putatively related to stress tolerance in *R. leucaenae* highlighted an intricated pattern comprising a variety of mechanisms that are probably orchestrated to tolerate the stressful conditions to which the strains are submitted on a daily basis. The capacity to synthesize Nod factors under abiotic stress might follow the same regulatory pathways as in CIAT 899^T^ and may help both to improve bacterial survival and to expand host range to guarantee the perpetuation of the symbiosis.

## Methods

### Bacterial strains and growth conditions

*R. leucaenae* strains CFN 299^T^ and CPAO 29.8 were obtained and are deposited at the “Diazotrophic and Plant Growth Promoting Bacteria Culture Collection of Embrapa Soja” (WFCC Collection # 1213, WDCM Collection # 1054), at Londrina, Paraná, Brazil. Several previous studies have reported properties of CFN 299^T^, culminating in its choice as the type strain of the *R. leucaenae* species [6]. Phenetic and genetic properties of CPAO 29.8 were also compiled before [6, 10]. Bacterial growth conditions and DNA extraction for genome sequencing were performed as described before [10].

### Sequencing, assembly and gap closure

*R. leucaenae* CFN 299^T^ whole-genome sequence reads were generated by MiSeq Illumina and 454 Roche (3 kb paired end library) sequencing at LNCC, Petropolis, Brazil. Hybrid *de novo* assemblies were generated using a combination of 454 and Illumina reads with the programs SPAdes [83] and Newbler (454 Life Sciences). Additionally, assemblies with only Illumina reads or only 454 reads were generated with the same programs. Raw Illumina reads were quality-trimmed before assembly using Trimmomatic [84]. Assemblies were merged using CISA [85], and then any possible misassemble was identified by read mapping with Bowtie [86] or Newbler. A set of primer pairs previously designed to close gaps in the symbiotic plasmid of *R. tropici* CIAT 899^T^ [11] was used to amplify gap regions in the symbiotic plasmid of *R. leucaenae* CFN 299^T^. PCR products were Sanger-sequenced and manually combined with the assembly using SeqMan Pro (DNASTAR Inc.).

Whole-genome sequences of *R. leucaenae* strain CPAO 29.8 were generated by MiSeq Illumina and 454 Roche (3 Kb paired end library) sequencing at LNCC, Petrópolis, Brazil. D*e novo* assembled were generated using Newbler.

A previously described strategy [11] to assign contigs to specific replicons was used. Chromosomal contigs were identified by mapping each sequence to closed chromosomes of other *Rhizobium* strains as these molecules are highly conserved in this genus. Likewise, contigs of the symbiotic plasmids were identified by sequence comparison with the previously reported and highly conserved tropici pSyms [11]. The smallest plasmid of CFN 299 was recovered as a single contig harboring *teu* genes previously mapped to that replicon.

### Annotation and genome comparisons

Gene prediction and annotation were performed using the Rapid Annotation using Subsystem Technology (RAST) server [87] using the following options: RAST gene caller, FIGfam release 70 for annotation, automatically error fixing, gap backfilling and no frameshift fixing. Genome sequences were aligned with BLASTN [88] with an E-value cutoff of 1e-5. Alignments were visualized with the Artemis comparison tool [89]. Orthologues were identified with PanOCT [90] using the results of all-versus-all alignments performed with BLASTP ’140].

### Symbiotic performance under regular and high temperature

A greenhouse experiment was performed with common bean cultivar Pérola (colored seeds) inoculated with eight main rhizobial strains microsymbionts of this legume: *R. leucaenae* CFN 299^T^ and CPAO 29.8, *R. tropici* CIAT 899^T^, *R. freirei* PRF 81^T^, *R. paranaense* PRF 35^T^, *R. etli* CFN 42^T^, *R. leguminosarum* bv. phaseoli TAL1121 and *R. gallicum* R602^T^. The experiment was performed in Leonard jars as described before [17] and inoculant preparation and seed inoculation were also performed as described before [17]. Non-inoculated controls without and with (70 mg N plant^−1^ week^−1^). Plants were grown at 28/23 or 35/23 °C (day/night). At early flowering stage, 30 days after seedling emergence, plants were harvested for the determination of nodulation (nodule number). At the same harvest shoot dry weight and concentration of N (N-Kjeldhal) in shoots were determined as described before [17], to estimate the value of total N accumulated in shoots.

### TLC analysis of nod factors

TLC analysis of Nod factors was performed as described before [15], in which Nod factors were labeled in vivo with 1 μCi of ^14^C-glucosamine hydrochloride (specific activity 52 mCi mmol^−1^) (Amersham Pharmacia Biotech, Buckinghamshire, England) at a final concentration of 1 μM in the medium and, after growth till exponential growth phase, extraction of the supernatant with water-saturated *n*-butanol and submitted to the TLC analysis. Synthesis of Nod factors was always verified in the presence or absence of flavonoids (apigenin, 3.7 μM). Abiotic stresses verified were temperature (28 and 37 °C), pH (5.0, 7.0, 9.0), and saline (up to 300 mM of NaCl). Growth conditions to verify the synthesis of Nod factors were as described before [13].

## Abbreviations

ABC, ATP-binding cassette; BNF, biological nitrogen fixation; CbG, cyclic beta glucans; CDS, coding sequence; COG, cluster of orthologous groups; HSP, heat-shock protein; LCOs, lipochitooligosaccharides; MFP, membrane fusion protein; MS, mechanosensitive; OHR, organic hydroperoxide; OMP, outer-membrane protein; RAST, rapid annotation using subsystem technology; ROS, reactive oxygen species; RTX, repeats in toxin; Sec, (general) secretion pathway; sHSP, small heat-shock protein; SOD, superoxide dismutase; T1SS, type-I secretion system; T4SS, Type-IV secretion system; T5SS, Type-V secretion system; Tat, twin-arginine translocation pathway; T-DNA, tumorigenic DNA; UDP, uridine diphosphate

## Additional files

Additional file 1: Table S1. Genome annotation of *Rhizobium leucaenae* strain CFN 299^T^ and correspondence with CDS of strain CPAO 29.8 and other related rhizobial genomes. Based on RAST and manual curation. (XLSX 3313 kb) Additional file 2: Table S2. Genome annotation of CDS related to stress tolerance in the genomes of *Rhizobium leucaenae* strains CFN 299^T^ and CPAO 29.8. Based on RAST and manual curation. (XLSX 17 kb) Additional file 3: Table S3. Symbiotic performance of *Rhizobium leucaenae* CFN 299^T^ and CPAO 29.8 under high temperature in comparison to other microsymbionts of common bean. Plants grown under greenhouse controlled condition, at 28/23 °C and 37/23 °C (day/night) and harvested at early flowering (30 days after seedling emergency). (DOCX 16 kb) Additional file 4: Table S4. Homology obtained in the comparison of nodulation genes of *R. leucaenae* CFN 299^T^ in comparison to strain CPAO 29.8 and *R. tropici* CIAT 899^T^. (DOCX 12 kb) Additional file 5: Table S5. Homology obtained in the comparison of nodulation genes of *R. leucaenae* CPAO 29.8 in comparison to strain CFN 299^T^ and *R. tropici* CIAT 899^T^. (DOCX 12 kb) Additional file 6: Figure S1. TLC analysis obtained with 14C labeled N-acetylglucosamine of Nod factors produced by rhizobial strains microsymbionts of *Phaseolus vulgaris* grown under acid (pH 4.0) or alkaline (pH 9.0) stress. Bacteria were induced (+) or not (−) with a flavonoid nod-gene inducer (apigenin, 3.7 μM). Profiles obtained for *R. tropici* CIAT 899^T^ were identical to those of *R. leucaenae* CFN 299^T^. (PPTX 1271 kb)
