# Supplementary material for: Genome of Rhizobium leucaenae strains CFN 299T and CPAO 29.8: searching for genes related to a successful symbiotic performance under stressful conditions
Source: BMC Genomics. 2016 Aug 2;17:534. doi: 10.1186/s12864-016-2859-z (PMC4971678; doi:10.1186/s12864-016-2859-z)
Supplement: Additional file 3: Table S3. — Symbiotic performance of Rhizobium leucaenae CFN 299T and CPAO 29.8 under high temperature in comparison to other microsymbionts of common bean. Plants grown under greenhouse controlled condition, at 28/23 °C and 37/23 °C (day/night) and harvested at early flowering (30 days after seedling emergency). (DOCX 16 kb) [file 12864_2016_2859_MOESM3_ESM.docx]

**Additional File 3: Table S3 Symbiotic performance of *Rhizobium leucaenae* CFN 299^T^ and CPAO 29.8 under high temperature in comparison to other microsymbionts of common bean.** Plants grown under greenhouse controlled condition, at 28/23ºC and 37/2 ºC (day/night) and harvested at early flowering (30 days after seedling emergency).

| **Species/Strain** | **t (°C)** | **Nodule Number**  (n° plant^-1^) | **Total N accumulated in shoots**  (mg N plant^-1^) |
| --- | --- | --- | --- |
| *R. leucaenae* CFN 299^T^ | 28ºC | 101 bc | 180 b |
|  | 35ºC | 75 d | 82 d |
| *R. leucaenae* CPAO 29.8 | 28ºC | 156 a | 218 a |
|  | 35ºC | 108 bc | 99 cd |
| *R. tropici* CIAT 899^T^ | 28ºC | 162 a | 222 a |
|  | 35ºC | 110 bc | 102 cd |
| *R. freirei* PRF 81^T^ | 28ºC | 142 a | 225 a |
|  | 35ºC | 90 cd | 98 cd |
| *R. paranaense* PRF35^T^ | 28ºC | 105 bc | 170 b |
|  | 35ºC | 78 d | 120 c |
| *R. etli* CFN 42^T^ | 28ºC | 118 b | 162 b |
|  | 35ºC | 35 e | 42 e |
| *R. leguminosarum* sv. phaseoli TAL1121 | 28ºC | 92 cd | 103 cd |
|  | 35ºC | 31 ef | 31 ef |
| *R. gallicum* R602^T^ | 28ºC | 35 e | 38 e |
|  | 35ºC | 10 f | 10 f |
| Non-inoculated – N-mineral | 28ºC | zero | 10 f |
|  | 35ºC | zero | 8 f |
| Non-inoculate + N-mineral | 35ºC | zero | 220 a |
|  | 35ºC | zero | 172 b |

Values represent the means of four replicates and when followed by the same letter, in the same column, do not show statistical difference by Tukey’s test (*p*≤0.05).

† N in shoot + roots + nodules
