# Supplementary material for: Genome of Rhizobium leucaenae strains CFN 299T and CPAO 29.8: searching for genes related to a successful symbiotic performance under stressful conditions
Source: BMC Genomics. 2016 Aug 2;17:534. doi: 10.1186/s12864-016-2859-z (PMC4971678; doi:10.1186/s12864-016-2859-z)
Supplement: Additional file 4: Table S4. — Homology obtained in the comparison of nodulation genes of R. leucaenae CFN 299T in comparison to strain CPAO 29.8 and R. tropici CIAT 899T. (DOCX 12 kb) [file 12864_2016_2859_MOESM4_ESM.docx]

**Additional File 4: Table S4 Homology obtained in the comparison of nodulation genes of *R. leucaenae* CFN 299^T^ in comparison to strain CPAO 29.8 and *R. tropici* CIAT 899^T^.**

| Genes | Localization^a^  (CFN 299^T^) | CIAT 899^T^ | | CPAO29.8 | |
| --- | --- | --- | --- | --- | --- |
|  |  | Identity | Coverage | Identity | Coverage |
| *nodD1* | 228347-227409 | 100% | 100% | 100% | 100% |
| *nodD2* | 184155-185093 | 100% | 100% | 100% | 100% |
| *nodD3* | 104782-103793 | 100% | 100% | 99.7% | 95.9% |
| *nodD4* | 279767-280753 | 100% | 100% | 100% | 100% |
| *nodD5* | 91115-92089 | 100% | 100% | 99.7% | 100% |
| *nodA1* | 228705-229253 | 100% | 92.9% | 100% | 92.9% |
| *nodA2* | 187663-188253 | 100% | 100% | 100% | 100% |
| *nodA3* | 105444-104896 | 100% | 92.9% | 100% | 92.9% |
| *nodB* | 229250-229909 | 100% | 100% | 100% | 100% |
| *nodC* | 229921-231279 | 100% | 100% | 100% | 100% |
| *nodS* (truncated) | 231730-231593 | 100% | 28.5% | 100% | 33.5% |
| *nodS* (complete) | 231710-231913 | 100% | 100% | 100% | 100% |
| *nodU* | 231949-233676 | 100% | 100% | 100% | 100% |
| *nodI* | 233664-234578 | 100% | 100% | 100% | 100% |
| *nodJ* | 234579-235367 | 100% | 100% | 100% | 100% |
| *nodH* | 235952-236701 | 100% | 100% | 100% | 100% |
| *nodP* | 236707-237606 | 100% | 100% | 100% | 100% |
| *nodQ1* | 237606-238424 | 100% | 100% | 100% | 100% |
| *nodQ2* | 238421-239503 | 100% | 100% | 100% | 100% |
| *hsnT* | 188426-190351 | 100% | 100% | 100% | 100% |
| *nodF* | 190447-190728 | 100% | 100% | 100% | 100% |
| *nodE* | 190729-191937 | 100% | 100% | 100% | 100% |
| *nodM* | 15586-13760 | 100% | 100% | 100% | 100% |

^a^ Localization in the genome of CFN 299^T^, according to the annotation obtained in RAST and displayed in Additional File 1: Table S1.
