# Supplementary material for: Genome of Rhizobium leucaenae strains CFN 299T and CPAO 29.8: searching for genes related to a successful symbiotic performance under stressful conditions
Source: BMC Genomics. 2016 Aug 2;17:534. doi: 10.1186/s12864-016-2859-z (PMC4971678; doi:10.1186/s12864-016-2859-z)
Supplement: Additional file 5: Table S5. — Homology obtained in the comparison of nodulation genes of R. leucaenae CPAO 29.8 in comparison to strain CFN 299T and R. tropici CIAT 899T. (DOCX 12 kb) [file 12864_2016_2859_MOESM5_ESM.docx]

**Additional File 5: Table S5 Homology obtained in the comparison of nodulation genes of *R. leucaenae* CPAO 29.8 in comparison to strain CFN 299^T^ and *R. tropici* CIAT 899^T^.**

| Genes | Range (CPAO29.8)^a^ | CIAT 899 | | CFN299 | |
| --- | --- | --- | --- | --- | --- |
|  |  | Identity | Coverage | Identity | Coverage |
| *nodD1* | 1222-296 | 100% | 100% | 100% | 98.7% |
| *nodD2* | 55094-56032 | 100% | 100% | 100% | 100% |
| *nodD^b^* | 1310-957 | 99.7% | 35.8% | 99.7% | 35.8% |
| *nodD^b^* | 4572-4207 | 99.7% | 35.5% | 99.7% | 35.5% |
| *nodD4* | 5905-6879 | 100% | 100% | 100% | 98.8% |
| *nodD5* | 6186-5212 | 99.7% | 100% | 99.7% | 100% |
| *nodA1* | 1550-2140 | 100% | 100% | 100% | 100% |
| *nodA2* | 58602-59192 | 100% | 100% | 100% | 100% |
| *nodA3* | 5276-4686 | 100% | 100% | 100% | 100% |
| *nodB* | 2137-2796 | 100% | 100% | 100% | 100% |
| *nodC* | 2808-4166 | 100% | 100% | 100% | 100% |
| *nodS* | 4192-4800 | 100% | 84.9% | 100% | 100% |
| *nodU* | 4836-6563 | 100% | 100% | 100% | 100% |
| *nodI* | 6551-7465 | 100% | 100% | 100% | 100% |
| *nodJ* | 7466-8254 | 100% | 100% | 100% | 100% |
| *nodH* | 8839-9588 | 100% | 100% | 100% | 100% |
| *nodP* | 9594-10493 | 100% | 100% | 100% | 100% |
| *nodQ1* | 10493-11311 | 100% | 100% | 100% | 100% |
| *nodQ2* | 11308-12390 | 100% | 100% | 100% | 100% |
| *hsnT* | 59365-61290 | 100% | 100% | 100% | 100% |
| *nodF* | 61386-61667 | 100% | 100% | 100% | 100% |
| *nodE* | 61668-62876 | 100% | 100% | 100% | 100% |
| *nodM* | 12711-14537 | 100% | 100% | 100% | 100% |

^a^ Localization in the genome of CPAO 29.8, according to the annotation obtained in RAST and displayed in Additional File 1: Table S1.

^b^ *nodD3* is interrupted by an IS21 family transposase
