# Supplementary material for: Genome of Rhizobium leucaenae strains CFN 299T and CPAO 29.8: searching for genes related to a successful symbiotic performance under stressful conditions
Source: BMC Genomics. 2016 Aug 2;17:534. doi: 10.1186/s12864-016-2859-z (PMC4971678; doi:10.1186/s12864-016-2859-z)
Supplement: Additional file 6: Figure S1. — TLC analysis obtained with 14C labeled N-acetylglucosamine of Nod factors produced by rhizobial strains microsymbionts of Phaseolus vulgaris grown under acid (pH 4.0) or alkaline (pH 9.0) stress. Bacteria were induced (+) or not (−) with a flavonoid nod-gene inducer (apigenin, 3.7 μM). Profiles obtained for R. tropici CIAT 899T were identical to those of R. leucaenae CFN 299T. (PPTX 1271 kb) [file 12864_2016_2859_MOESM6_ESM.pptx]

## Slide 1
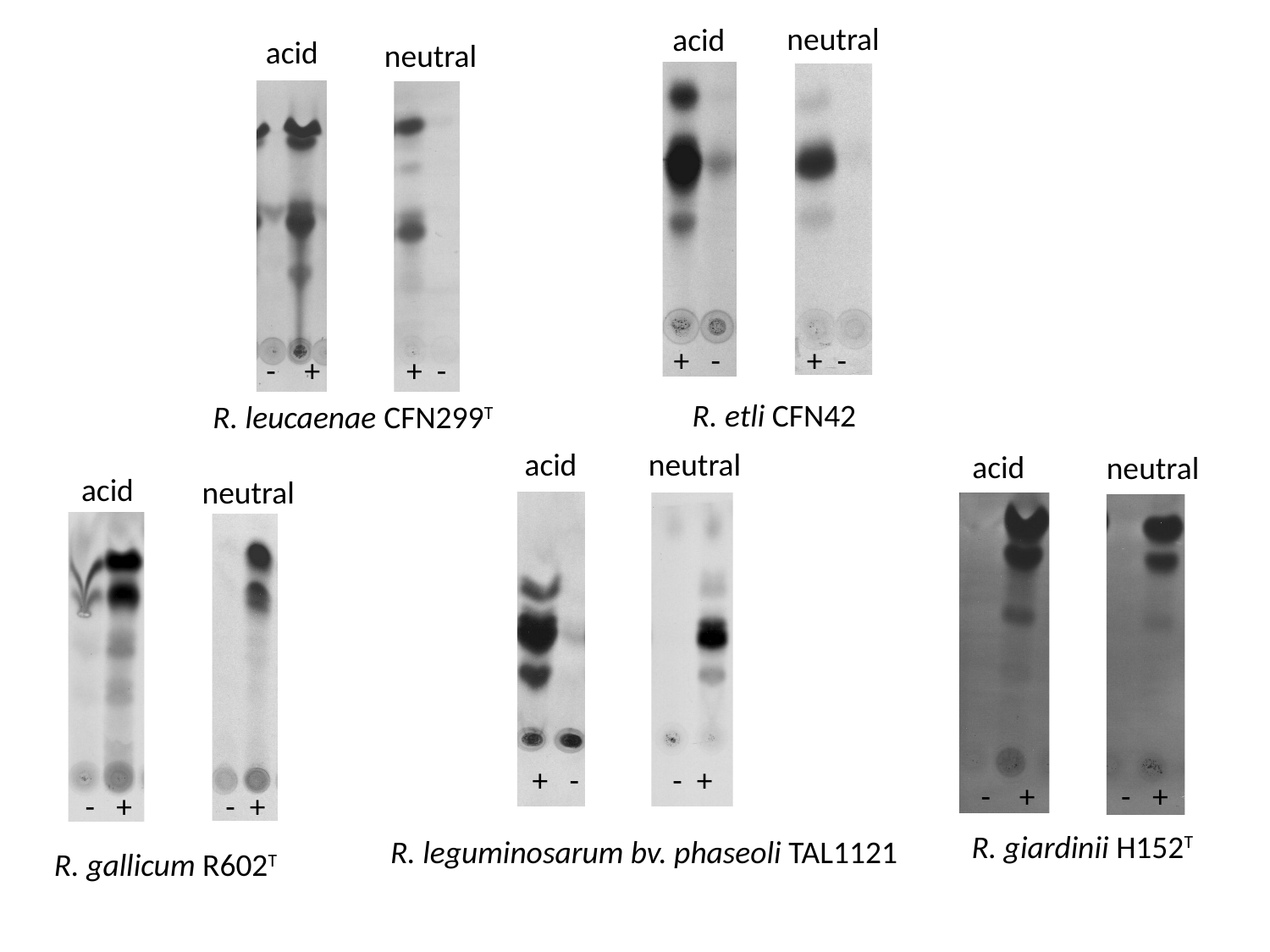

neutral
acid
acid
neutral
+ - + -
- + + -
R. etli CFN42
R. leucaenae CFN299T
acid
neutral
acid
 neutral
acid
 neutral
+ - - +
- + - +
- + - +
R. giardinii H152T
R. leguminosarum bv. phaseoli TAL1121
R. gallicum R602T
